# A Risk Model for Prognosis and Treatment Response Prediction in Colon Adenocarcinoma Based on Genes Associated with the Characteristics of the Epithelial-Mesenchymal Transition

**DOI:** 10.3390/ijms241713206

**Published:** 2023-08-25

**Authors:** Hongyu Huang, Tianyou Li, Ziqi Meng, Xueqian Zhang, Shanshan Jiang, Mengying Suo, Na Li

**Affiliations:** 1 School of Medicine, Nankai University, 94 Weijin Road, Tianjin 300071, China; 2 Tianjin Key Laboratory of Tumor Microenvironment and Neurovascular Regulation, Tianjin 300071, China

**Keywords:** CRC, EMT, multigene prognosis-related risk model, clinical significance, therapeutic response

## Abstract

The epithelial-mesenchymal transition (EMT) is an important process during metastasis in various tumors, including colorectal cancer (CRC). Thus, the study of its characteristics and related genes is of great significance for CRC treatment. In this study, 26 EMT-related gene sets were used to score each sample from The Cancer Genome Atlas program (TCGA) colon adenocarcinoma (COAD) database. Based on the 26 EMT enrichment scores for each sample, we performed unsupervised cluster analysis and classified the TCGA-COAD samples into three EMT clusters. Then, weighted gene co-expression network analysis (WGCNA) was used to investigate the gene modules that were significantly associated with these three EMT clusters. Two gene modules that were strongly positively correlated with the EMT cluster 2 (worst prognosis) were subjected to Cox regression and least absolute shrinkage and selection operator (LASSO) regression analysis. Then, a prognosis-related risk model composed of three hub genes *GPRC5B*, *LSAMP*, and *PDGFRA* was established. The TCGA rectal adenocarcinoma (READ) dataset and a CRC dataset from the Gene Expression Omnibus (GEO) were used as the validation sets. A novel nomogram that incorporated the risk model and clinicopathological features was developed to predict the clinical outcomes of the COAD patients. The risk model served as an independent prognostic factor. It showed good predictive power for overall survival (OS), immunotherapy efficacy, and drug sensitivity in the COAD patients. Our study provides a comprehensive evaluation of the clinical relevance of this three-gene risk model for COAD patients and a deeper understanding of the role of EMT-related genes in COAD.

## 1. Introduction

According to the statistics, colorectal cancer (CRC) is a common cause of cancer death in both men and women [1], and its incidence is increasing worldwide [2]. CRC treatment has recently improved, and with new treatment regimens, patients suffering from advanced CRC have nearly doubled their overall survival (OS) [3]. The late onset of CRC symptoms makes it crucial to investigate more effective clinical treatments and antitumor strategies. Therefore, it is necessary to construct efficient gene models to improve the prediction efficacy for survival and the sensitivity to antitumor drugs in CRC patients [4].

The epithelial-mesenchymal transition (EMT), a significant phenotype in cancer metastasis, is a cellular process in which epithelial cells obtain a mesenchymal phenotype, downregulate epithelial cell function, reduce apoptosis, and promote immunosuppression in tumor cells [5]. During the EMT, epithelial properties are suppressed, and mesenchymal properties emerge through complex mechanisms, such as gene expression and post-translational regulation [6]. The cells are detached from the basement membrane and obtain increased mobility and migratory capacity, showing the characteristics of advanced malignancy. The EMT has a considerable influence on tumor development, prognosis, and the outcomes from chemotherapy and immunotherapy [7], making it an important target for anti-cancer therapy [8]. The EMT gene network is significantly related to poor prognosis and anti-PD-1 resistance [9]. Although several studies have been conducted to analyze the prognostic genes involved in the EMT, the systematic modeling of prognostic-related risks and the analysis of their clinical significance are still inadequate.

Based on gene set variation analysis (GSVA) of 26 EMT gene sets, we divided the samples from The Cancer Genome Atlas program (TCGA) colon adenocarcinoma (COAD) database into three clusters. Then, we used weighted gene co-expression network analysis (WGCNA) to identify the genes most relevant to these clusters. Based on a survival analysis, we established a multi-gene prognosis-related risk model, and we analyzed the correlation between our risk model and the tumor microenvironment (TME), the tumor mutation burden (TMB), tumor metastasis, immune infiltration, the immunotherapy effect, and drug sensitivity. This study adds to the basic research of related prognostic genes and provides a new pathway for discovering novel therapeutic targets for COAD.

## 2. Results

### 2.1. Patients in Different EMT Subtypes Showed Different Prognoses and EMT Characteristics

**Figure 1:**
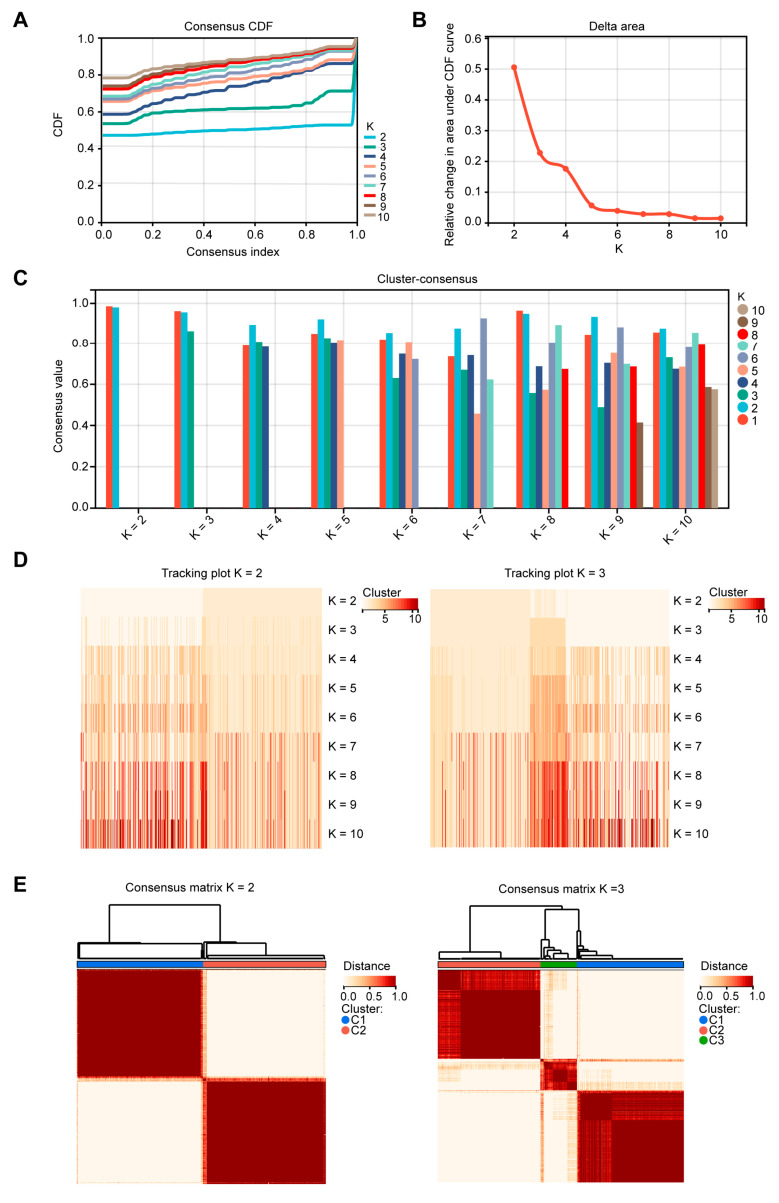
Unsupervised clustering was performed based on the 26 epithelial-mesenchymal transition (EMT) enrichment scores for each colon adenocarcinoma (COAD) sample. (**A**) Cumulative distribution function (CDF) diagram of the consensus distribution for each K; (**B**) delta area plot of the relative change in area under the CDF curve for each K; (**C**) histogram of the consensus value of each K ranged from K = 2 to K = 10; (**D**) tracking plot of the classification and subtypes of samples from K = 2 to K = 10; (**E**) consensus clustering heatmap showing the clustering situation of the COAD samples with different clustering numbers (K = 2 and 3).

**Figure 2:**
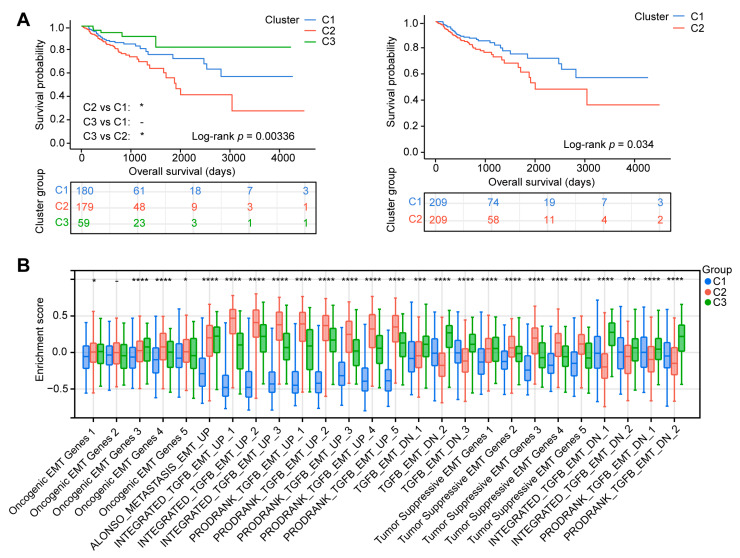
Characterization and prognosis of different EMT clusters. (**A**) Kaplan–Meier overall survival (OS) curves for The Cancer Genome Atlas program (TCGA) COAD patients categorized as different EMT clusters (K = 2 and 3). (**B**) Box plot showing the differences in 26 EMT enrichment scores calculated using the gene set variation analysis (GSVA) algorithm among the three clusters. Kruskal–Wallis test; -, no significance, * *p* < 0.05, *** *p* < 0.001, **** *p* < 0.0001.

Based on the 26 EMT gene sets (Figure S1A–D, Tables S1 and S2), we employed the R package from GSVA to obtain 26 EMT enrichment scores for each TCGA-COAD sample. Using the enrichment scores of each sample, we performed cluster analysis by employing ConsensusClusterPlus. The cumulative distribution function (CDF) diagram displayed the consensus distribution of each K (Figure 1A). The delta area plot showed the relative differences in the CDF curve area (Figure 1B). According to the intra-group average consistency evaluation, K = 2 ranked highest, followed by K = 3 (Figure 1C). The tracking plot showed that the classification was stable when there were two or three clusters (Figure 1D). Accordingly, the COAD patients were categorized into two and three distinct clusters (Figure 1E). When K = 3, the OS of the COAD patients in cluster 2 (179 patients) was lower than that in clusters 1 (180 patients) and 3 (59 patients). When K = 2, the OS of the COAD patients in cluster 2 (209 patients) was lower than that in cluster 1 (209 patients) (Figure 2A). Then, the characteristics of the three EMT clusters were further analyzed. The highest enrichment scores of many oncogenic EMT gene sets and EMT-upregulated gene sets were observed in cluster 2, including Oncogenic EMT Genes 4, INTEGRATED_TGFB_EMT_UP_1 to 3, and PRODRANK_TGFB_EMT_UP_1 to 5. The highest enrichment scores of many EMT-downregulated gene sets were observed in cluster 3, including gene sets TGFB_EMT_DN_1 to 3, INTEGRATED_TGFB_EMT_DN_1, and PRODRANK_TGFB_EMT_DN_2 (Figure 2B).

### 2.2. Independent Analysis to Verify the Function of the Three EMT Clusters

To further validate the classification of the three EMT clusters derived from the unsupervised clustering of the 26 EMT gene sets, we conducted an independent cluster analysis by employing ConsensusClusterPlus utilizing the expression profile of the 33 CRC EMT markers. The TCGA-COAD samples were categorized into two subtypes (A1 and A2). The principal component analysis (PCA) showed obvious differences between A1 and A2 (Figure S2A). The Kaplan–Meier analysis showed that the COAD patients of A2 (212 patients) had worse OS than those of A1 (206 patients) (Figure S2B). The heatmap and submap revealed that C1 significantly correlated with A1 (*p* = 1.0 × 10^−3^) and that C2 significantly correlated with A2 (*p* = 1.0 × 10^−3^) (Figure S2C,D). Between the two EMT clusters, A2 was enriched with the oncogenic EMT gene sets and EMT-upregulated gene sets, and A1 was enriched with many EMT-downregulated gene sets (Figure S2E). This independent analysis thus verified the function of the three EMT clusters derived from the 26 EMT gene sets.

### 2.3. Identification of Modules and Hub Genes Associated with EMT Cluster 2 by WGCNA

**Figure 3:**
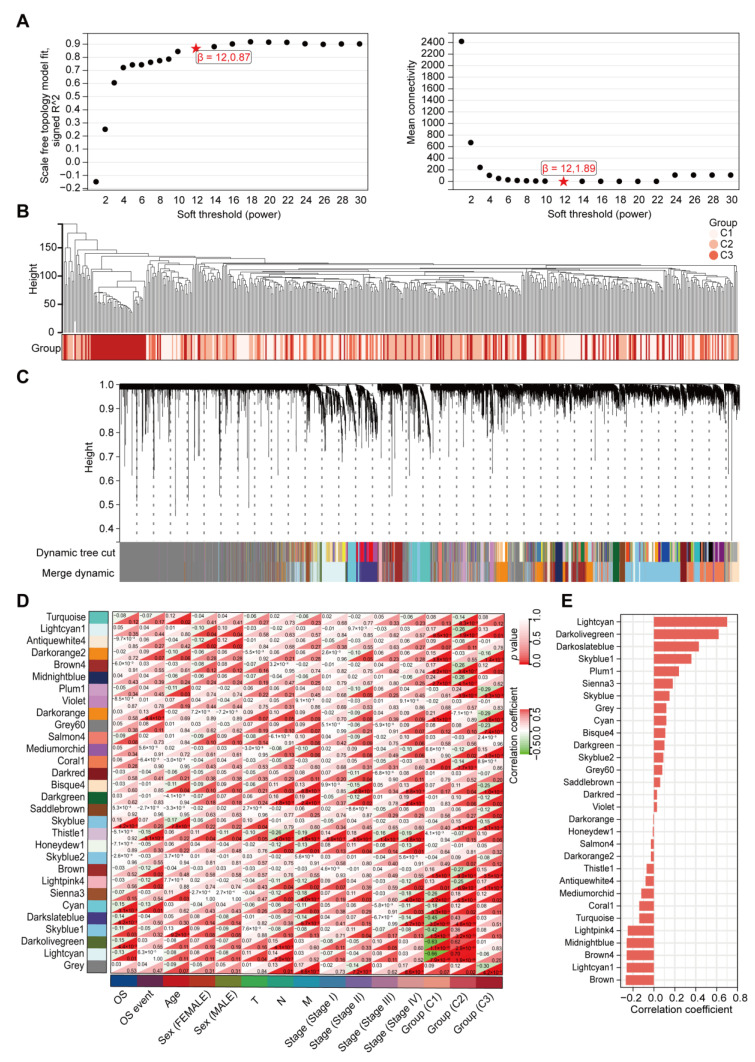
Identification of EMT cluster-associated modules via weighted gene co-expression network analysis (WGCNA) in the TCGA-COAD dataset. (**A**) Scale independence and mean connectivity of the different soft thresholds for determining the optimal soft threshold. (**B**) Sample dendrogram with the heatmap of the EMT subtypes. (**C**) Cluster tree showing that after hierarchical clustering and module merging of the genes, 30 co-expression modules with different colors were obtained. (**D**) Heatmap showing the relevance of the 30 co-expression modules with clinical features and three EMT clusters. (**E**) Histogram showing the correlation between the gene modules and EMT cluster 2.

We then applied WGCNA to determine the gene modules related to EMT in the TCGA-COAD dataset. First, scale independence and mean connectivity were plotted to determine 12 as the optimal soft threshold (β) (Figure 3A). We applied the Pearson correlation coefficient pair to the cluster samples and drew the sample clustering plot (Figure 3B). We set the minimum size of the module to 30 and the sensitivity to 4. After hierarchical clustering and the merging of some modules, we obtained 30 co-expression modules (Figure 3C). After we compared the 30 modules, we selected the lightcyan and darkolivegreen modules for further study because they had the strongest positive correlation with cluster 2 (lightcyan: correlation coefficient = 0.70, *p* = 1.9 × 10^−56^; darkolivegreen: correlation coefficient = 0.62, *p* = 2.9 × 10^−41^); the strongest inverse correlation with the cluster 1 subtype (lightcyan: correlation coefficient = −0.66, *p* = 7.9 × 10^−48^; darkolivegreen: correlation coefficient = −0.63, *p* = 1.1 × 10^−42^); and an inverse correlation with OS (Figure 3D,E).

### 2.4. GO and KEGG Analyses of Selected Module Genes and PPI Network Construction

**Figure 4:**
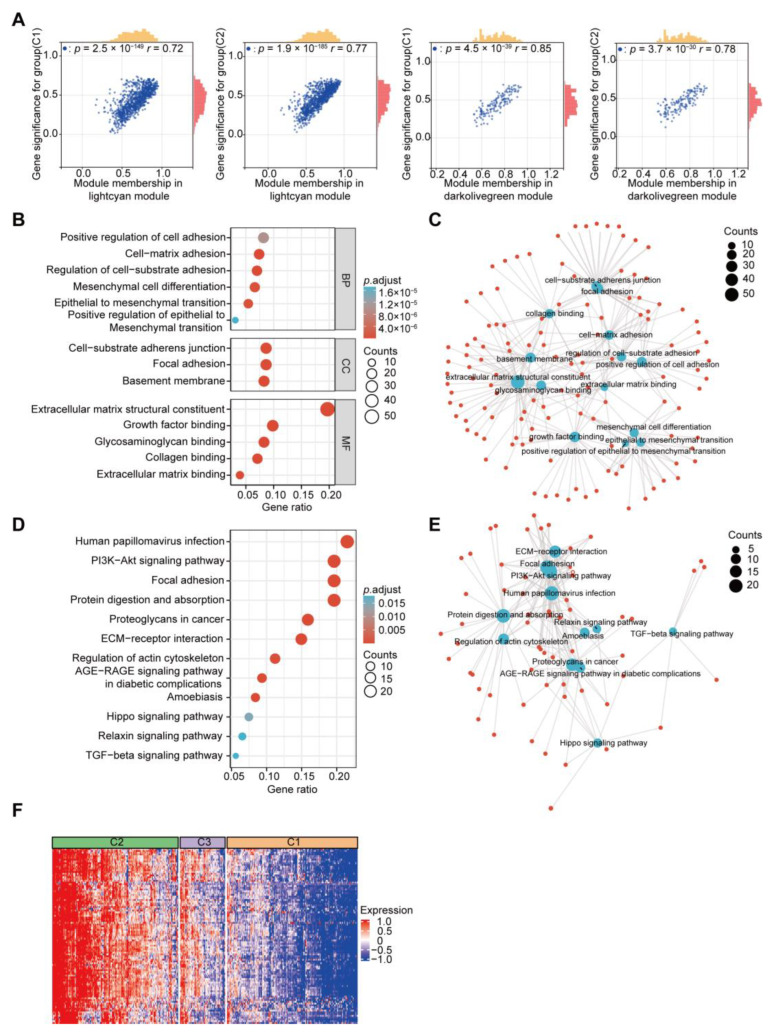
Identification of the gene modules associated with EMT clusters 1 and 2. (**A**) Scatter diagram showing the relevance between module membership in the lightcyan and darkolivegreen modules with gene significance for cluster C1 and cluster C2. (**B**) Bubble plot showing Gene Ontology (GO) terms, such as biological process (BP), cellular component (CC), and molecular function (MF) associated with the genes in the lightcyan and darkolivegreen modules. (**C**) Network diagram showing the connection between these GO terms. The red dots represent the module genes, and the blue dots represent the GO terms. (**D**) Bubble plot showing the Kyoto Encyclopedia of Genes and Genomes (KEGG) pathways associated with the genes in the lightcyan and darkolivegreen modules. (**E**) Network diagram of the connection between KEGG pathways. The red dots represent the module genes, and the blue dots represent the KEGG pathways. (**F**) Heatmaps showing the genes from the lightcyan and darkolivegreen modules among the different EMT subtypes.

Following the parameter standards of module membership (MM) > 0.65 and gene significance (GS) > 0.4, we finally obtained 585 candidate hub genes from the two modules (Figure 4A). To determine the functions and related pathways of the 585 genes, we conducted Gene Ontology (GO) and Kyoto Encyclopedia of Genes and Genomes (KEGG) analyses. The main enriched GO terms included positive regulation of cell adhesion, mesenchymal cell differentiation, EMT, focal adhesion, growth factor binding, and collagen binding (Figure 4B,C). According to the KEGG analysis, the two modules were primarily associated with the PI3K-Akt signaling pathway, focal adhesion, proteoglycans in cancer, and the TGF-β signaling pathway (Figure 4D,E). The heatmap analysis showed that the expression of the modular genes increased in cluster 2 and decreased in cluster 1 (Figure 4F). By applying the STRING database, we built a protein–protein interaction (PPI) network and visualized the interactions between the module genes (Figure S3).

### 2.5. Construction of the Prognostic Risk Score Model Based on the Hub Genes

**Figure 5:**
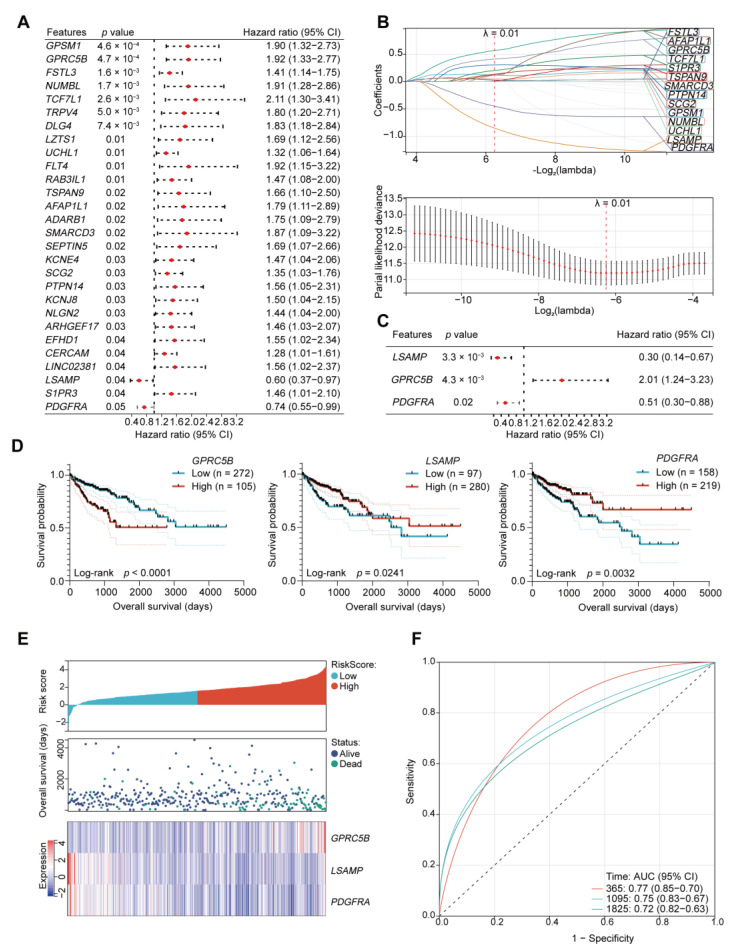
Construction of the risk score model. (**A**) Univariate Cox regression analysis identifying genes significantly relevant to OS based on genes from the lightcyan and darkolivegreen modules. (**B**) Least absolute shrinkage and selection operator (LASSO) regression analysis for further screening of the important genes. (**C**) Genes significantly relevant to OS were screened out via multivariate Cox analysis. (**D**) Kaplan–Meier OS curves of the patients with high and low expression of *GPRC5B*, *LSAMP*, and *PDGFRA*, shown with 95% confidence intervals. (**E**) Expression heatmap of the three hub genes with the OS status and risk score of each patient. (**F**) 1-, 3-, and 5-year receiver operating characteristic (ROC) curves for OS prediction by the risk score model in TCGA-COAD patients.

By analyzing data from the TCGA-COAD dataset, we identified 28 module genes with significant correlation with OS via univariate Cox analysis (Figure 5A). Then, we implemented least absolute shrinkage and selection operator (LASSO) regression analysis and filtered out 14 genes with the Lambda value set to 0.01316 (Figure 5B). Using multivariate Cox regression analysis, we eventually selected *LSAMP*, *GPRC5B*, and *PDGFRA*, which had the highest prognostic value among the 14 genes (*p* < 0.05) (Figure 5C). According to the hazard ratio (HR) of the three genes, *GPRC5B* was considered a risk factor of OS, while *LSAMP* and *PDGFRA* were considered protective factors. The Kaplan–Meier curves demonstrated the distinct OS outcomes between the groups with high and low expression of these genes, respectively (Figure 5D). The patients with high *GPRC5B* expression had worse OS, and the patients with high *LSAMP* and *PDGFRA* expression had better OS. Then, we established a prognostic risk score model to predict the patients’ OS. In the scatter plot, the death samples accumulated as the risk scores increased (Figure 5E). The receiver operating characteristic (ROC) curves demonstrated the reliability of the prognostic risk score model (AUC (95%CI)): 365 days: 0.77 (0.85–0.70); 1095 days: 0.75 (0.83–0.67); 1825 days: 0.72 (0.82–0.63) (Figure 5F).

### 2.6. Further Validation of the Prognostic Value of the Risk Score Model

**Figure 6:**
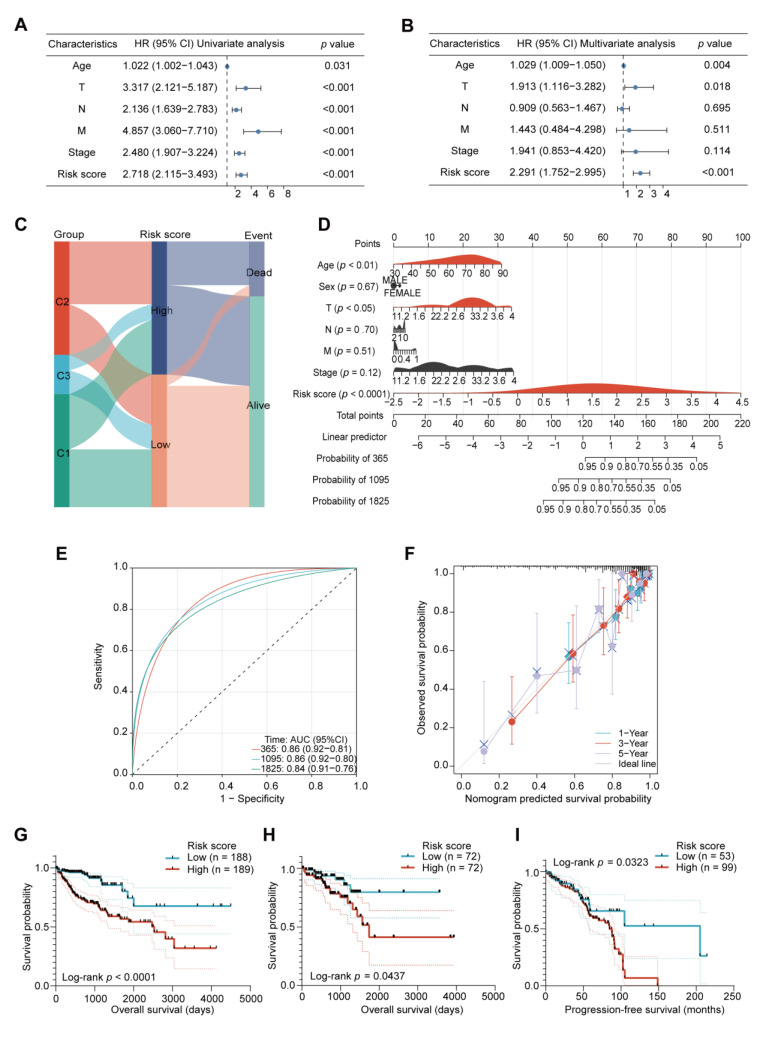
Validation of the risk score-based prognostic model. (**A**) Univariate Cox analysis of risk score and clinicopathological features. (**B**) Multivariate Cox analysis of risk score and clinicopathological characteristics. (**C**) Alluvial diagram displaying the distributions of COAD patients in the three EMT clusters across the two risk groups and two OS statuses. (**D**) Nomogram for prediction of patient OS using risk scores and clinicopathological characteristics. (**E**) 1-, 3-, and 5-year ROC curves for the OS prediction by nomogram in TCGA-COAD patients. (**F**) 1-, 3-, and 5-year calibration plot for the OS prediction by nomogram in TCGA-COAD patients. (**G**) Kaplan–Meier OS curves for the high- and low-risk groups from the TCGA-COAD dataset, shown with 95% confidence intervals. (**H**) Kaplan–Meier OS curves for the high- and low-risk groups from the TCGA rectal adenocarcinoma (READ) dataset, shown with 95% confidence intervals. (**I**) Kaplan–Meier progression-free survival (PFS) curves for the high- and low-risk groups from the GSE103479 dataset, which includes colorectal cancer (CRC) patients who received adjuvant 5-fluorouracil (5FU)-based chemotherapy, shown with 95% confidence intervals.

To test the reliability of our risk model, both univariate and multivariate Cox analyses were performed, confirming that the risk score was an independent predictor of COAD patient survival (Figure 6A,B). The alluvial diagram further revealed that the patients with high-risk scores showed a larger proportion of death outcomes than those with low-risk scores (Figure 6C). Then, we rendered a nomogram to predict the 1-, 3-, and 5-year OS of the COAD patients according to their risk scores and clinicopathological features (Figure 6D). The ROC curves and calibration plots confirmed that the nomogram could effectively predict the OS of the COAD patients (Figure 6E,F). The Kaplan–Meier analysis revealed that patients with high-risk scores had worse prognoses (Figure 6G). The same tendency was also observed in the TCGA rectal adenocarcinoma (READ) and GSE103479 datasets (Figure 6H,I). Therefore, the risk score model based on the three hub genes had good prognostic value for predicting the prognosis of patients with CRC.

### 2.7. Immune Infiltrations and Mutation Landscape in the Two Risk Groups

**Figure 7:**
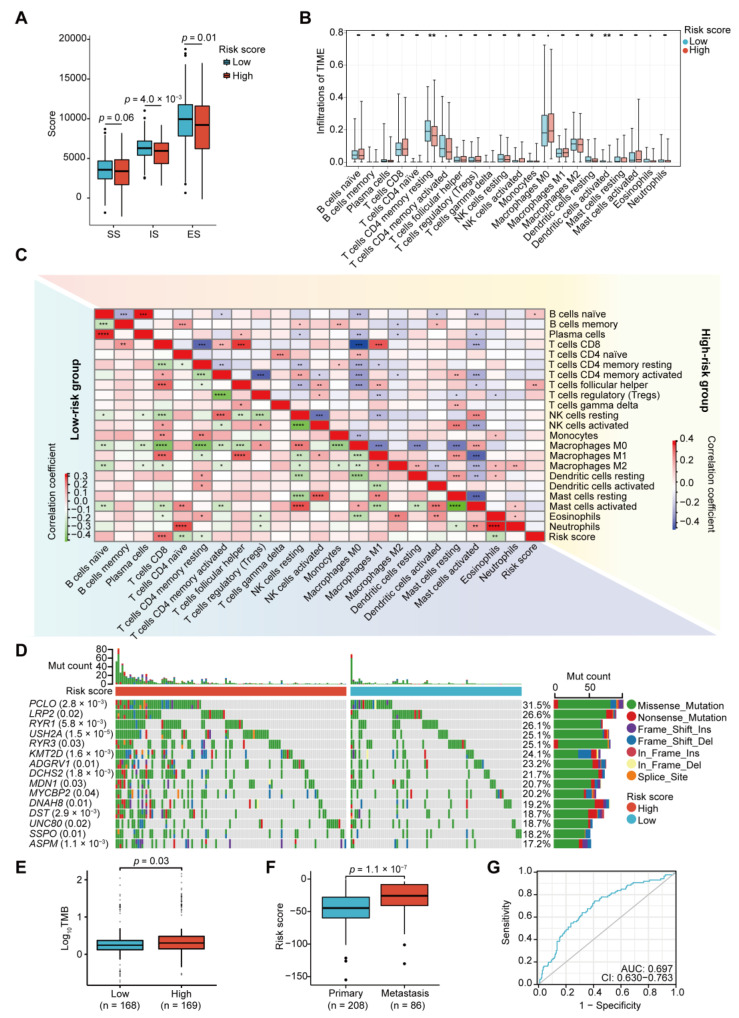
Exploration of immune infiltration and tumor mutation burden (TMB) based on the risk score. (**A**) Box plot of the stromal scores (SS), immune scores (IS), and Estimation of STromal and Immune cells in MAlignant Tumor tissues using Expression data (ESTIMATE) scores (ES) for the two risk groups (Wilcoxon test). (**B**) The infiltrations of immune cells in the tumor immune microenvironment (TIME) are compared between the two risk groups. The data are presented as box plots. Wilcoxon test; -, no significance; * *p* < 0.05; ** *p* < 0.01; *** *p* < 0.001, **** *p* < 0.0001. (**C**) Correlations between the risk score and infiltration of the immune cells in the two risk groups. (**D**) Mutational landscape of the top 15 genes associated with the risk score. (**E**) Box plot of TMB scores in the two risk groups from the TCGA-COAD dataset (Wilcoxon test). (**F**) Risk scores of liver metastasis and primary samples of CRC from the GSE81423, GSE81558 and GSE41258 datasets. The data are presented as box plots (Wilcoxon test). (**G**) ROC curve for the liver-metastasis prediction by risk score model in the GSE81423, GSE81558 and GSE41258 datasets.

In the two risk groups, the high-risk group exhibited a lower Estimation of STromal and Immune cells in MAlignant Tumor tissues using Expression data (ESTIMATE) score, indicating its higher tumor purity (Figure 7A). According to Cell-type Identification by Estimating Relative Subsets of RNA Transcripts (CIBERSORT), the high-risk group showed lower infiltrations of resting memory CD4 T cells and dendritic cells (Figure 7B). We analyzed the correlation coefficient of the risk score and the infiltrations of the immune cells of the samples in both the low- and high-risk groups. The risk score was positively correlated with CD8 T cells and inversely correlated with naïve CD4 T cells, resting memory CD4 T cells, and eosinophils in the low-risk group. In the high-risk group, the risk score had positive correlations with follicular helper T cells and naïve B cells (Figure 7C).

In the mutational panorama, the top 15 genes with the high mutation frequencies and significant differences between the high-risk and low-risk groups were displayed, including *PCLO*, *LRP2*, *RYR1*, and *USH2A* (Figure 7D). Moreover, the TMB score of the high-risk group was higher than that of the low-risk group (Figure 7E).

In addition, the metastatic samples had a higher risk score than the primary samples, suggesting a correlation between the risk score and tumor metastasis (Figure 7F). Moreover, the ROC curve demonstrated the reliability of the risk score in predicting liver metastasis (AUC (95% CI): 0.697 (0.630–0.763), Figure 7G).

### 2.8. Risk Model Prediction of Drug Sensitivity and Immunotherapy Response

**Figure 8:**
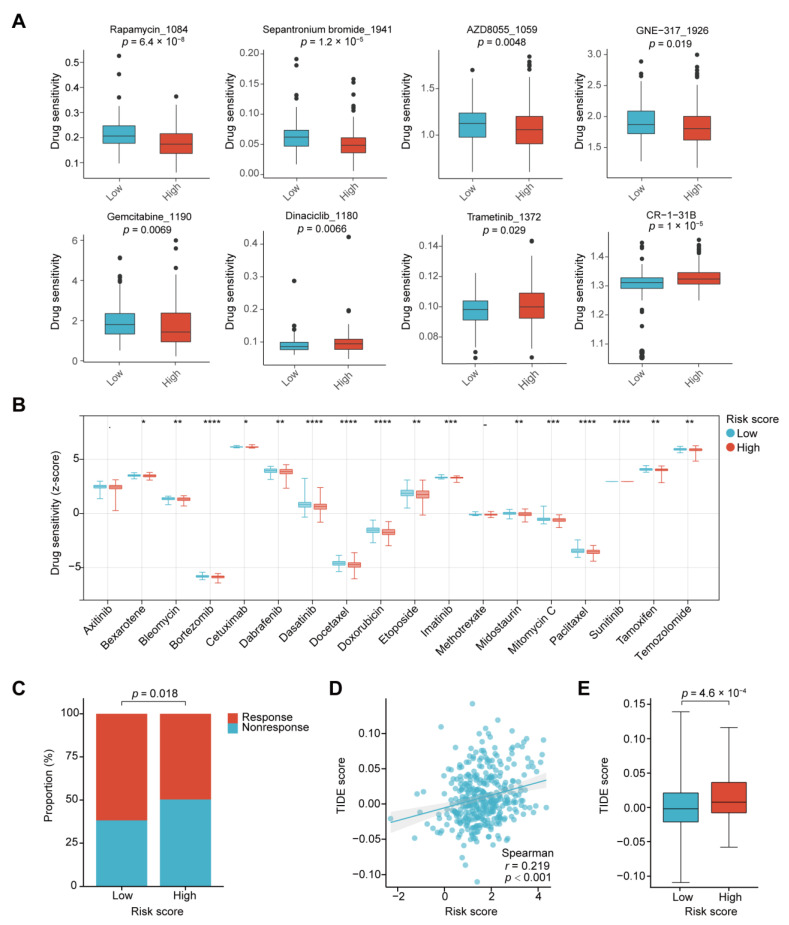
Value of the risk score for predicting drug sensitivity and immunotherapy response. (**A**) Drug sensitivity in the high- and low-risk groups was estimated using the oncoPredict algorithm in the TCGA-COAD dataset. The data are presented as box plots (Wilcoxon test). (**B**) Drug sensitivity in high- and low-risk groups were estimated using the pRRophetic algorithm. The data are presented as box plots. Wilcoxon test; -, no significance, * *p* < 0.05, ** *p* < 0.01, *** *p* < 0.001, **** *p* < 0.0001. (**C**) Distributions of responders and non-responders to immunotherapy in the high- and low-risk groups from the TCGA-COAD dataset estimated from the TIDE algorithm (χ^2^ test). (**D**) Scatter plot showing the correlation between the TIDE score and the risk score of the TCGA-COAD samples. (**E**) Box plot of TIDE scores in the low- and high-risk groups from the TCGA-COAD dataset (Wilcoxon test).

To investigate the ability of our risk score model to predict drug sensitivity, we compared drug sensitivity using the oncoPredict algorithm between the two risk clusters. As shown in Figure 8A, the sensitivities to rapamycin_1084, sepantronium bromide_1941, AZD8055_1059, GNE–317_1926, and gemcitabine_1190 were considerably higher in the low-risk group. However, the high-risk group was more sensitive to dinaciclib_1180, trametinib_1372, and CR–1–31B. By employing the pRRophetic R package, we also identified other drugs that were more sensitive in the low-risk group, such as dabrafenib and temozolomide (Figure 8B). Using the Tumor Immune Dysfunction and Exclusion (TIDE) algorithm, we then analyzed the correlation between the risk score and the immunotherapy response. As shown in Figure 8C, there was a smaller proportion of patients with the estimated response to immunotherapy in the high-risk group (91 of 183, 49.7%) than in the low-risk group (120 of 193, 62.2%). The risk score was positively correlated with the TIDE score, and the TIDE score of the high-risk group was significantly higher than that of the low-risk group (Figure 8D,E). These results indicate that the high-risk group had poor response to immunotherapy. These findings demonstrated the value of the risk score model for choosing possible drug and immunotherapy strategies for patients with COAD.

### 2.9. GSEA Analysis of Gene Sets Enriched in High- and Low-Risk Groups

Based on the results of a GSEA analysis of GO biological process (GOBP) gene sets, we found that the gene sets of complement activation, positive regulation of interferon alpha production, and natural killer cell proliferation were significantly enriched in the low-risk group. The gene set of regulation of the apoptotic process involved in the development was significantly enriched in the high-risk group (Figure S4).

## 3. Discussion

EMT is an important biological process in which malignant tumor cells derived from epithelial cells have acquired migration and invasion abilities [10]. It is an important therapeutic target in cancers and plays a critical role in therapeutic resistance [11]. This study, to our knowledge, presents the first systematic bioinformatic analyses of large-scale COAD cohorts with 26 EMT gene sets. In this study, precise molecular subtyping of the EMT characteristics was used to stratify CRC patients. The identification of the gene expression patterns associated with the EMT subtypes based on this grouping method provides a reference for the development of more effective personalized treatment plans for patients.

According to a series of survival analyses, three genes (*LSAMP*, *GPRC5B*, and *PDGFRA*) were eventually screened out to construct a risk score model. This risk score model showed high efficacy in predicting the survival of COAD patients. Considering the potential limitations of the risk model based only on the TCGA-COAD dataset, we conducted validation studies on the TCGA-READ dataset and GSE103479 to confirm the clinical relevance of the risk score in CRC.

Moreover, the risk score is associated with the infiltration of certain types of immune cells, and the TIDE scores differ between the two risk groups, indicating our risk model’s value for predicting a patient’s response to immunotherapy. However, because publicly available transcriptomic data of CRC patients that undergo immunotherapy are currently quite limited, future evaluations of the actual relationship between the risk groups and the responsiveness to CRC immunotherapy are required.

Another important finding of our study is the correlation between the estimated sensitivity to antitumor drugs and the risk score of COAD patients. Some drugs were predicted to have improved therapeutic effects on patients who had high risk scores, while others showed better therapeutic effects on patients who had low risk scores. Notably, some of these drugs were previously validated for their therapeutic effect on CRC. For example, an optimized cocktail of axitinib, erlotinib, and dasatinib induced apoptosis in CRC cell lines [12]. Additionally, cetuximab gained approval as a first-line treatment for patients with EGFR-expressing, RAS wild-type metastatic CRC, particularly in cases where chemotherapy has failed or is not well tolerated [13]. However, some of the drugs associated with the risk score were primarily used for other types of cancers or medical conditions. In this case, we aim to establish potential connections for drug repurposing for CRC treatment. Overall, our findings suggest possibilities for personalized treatment approaches and highlight the potential for repurposing existing drugs to enhance therapeutic outcomes for CRC patients. However, further investigations and clinical trials are necessary to validate the efficacy and safety of these candidate drugs in CRC treatment.

Although there have been studies on EMT-related genes and their clinical significance in CRC, our research has the following innovative points. First, we did not directly use the existing EMT gene sets to construct the prognostic model. Instead, we performed consensus clustering based on the GSVA enrichment scores of 26 EMT gene sets and used WGCNA to find the gene modules. Therefore, the genes used for the construction of our prognostic model were correlated with distinct EMT-related subgroups. Second, we established a novel risk model based on three genes and investigated the clinical significance of our risk score for predicting survival, immune infiltrations, somatic mutations, metastasis, sensitivity to therapeutic drugs, and immunotherapy.

However, this study has certain limitations. First, while we diligently included patients from diverse age groups, genders, races, and pathological stages from several public datasets, we acknowledge that the diversity and comprehensiveness of our cohort may still be insufficient. Addressing this limitation by expanding the demographic representation could bolster the robustness and applicability of our findings. Second, the construction of our prognostic model relied on the analyses of publicly available datasets, potentially leading to the omission of certain relevant clinical nuances. Additionally, inherent biases within the data and algorithms utilized must be acknowledged as these may affect the accuracy of the prognostic model.

Notwithstanding these limitations, we anticipate that our findings will lay the foundation for elucidating the specific mechanisms by which the three genes affect the progression of CRC. Furthermore, the model’s clinical utility can be explored from various perspectives, including immunotherapy, drug repurposing, and other innovative research fields. Meanwhile, this study contributes to the development of personalized treatment strategies for COAD.

## 4. Materials and Methods

### 4.1. Data Collection and Processing

The Cancer Genome Atlas program (TCGA) (colon adenocarcinoma (COAD) and rectal adenocarcinoma (READ)) and the Gene Expression Omnibus (GEO) datasets (GSE103479, GSE81423, GSE81558, and GSE41258), with their corresponding clinical and survival notes, were downloaded from the TCGA (https://portal.gdc.cancer.gov/, (accessed on 4 September 2022)) and GEO databases (https://www.ncbi.nlm.nih.gov/geo/, (accessed on 7 September 2022)), respectively. The data on gene expression were preprocessed. First, rows (genes) with a 0 ratio greater than 50% were removed. Furthermore, the “impute.KNN” function of the R package impute (v 1.72.3) was used, and the number of neighbors was set to 10 to complete the missing data. Then, log2(x + 1) was used to transform the data. The R package stats (v 3.6.0) was used for principal component analysis (PCA). Specifically, we first calculated the z-score for the expression profile and then used the prcomp function for the dimensionality reduction analysis to obtain the matrix. As a result, 447 of 458 COAD and 144 of 167 READ samples were retained for the study after preprocessing and filtering. For the CRC database GSE103479, 152 of 156 samples were retained.

To explore the correlation between colorectal liver metastasis and the risk score, we obtained samples from the GSE81423, GSE81558 and GSE41258 datasets, including 129 colorectal liver metastases and 209 primary colorectal adenocarcinomas. Then, we merged them into one expression profile using the inSilicoMerging R package [14] (v 1.12.0) and removed the batch effect using the empirical Bayes methods [15] of sva R package (v 3.46.0); 294 samples remained.

### 4.2. EMT Signatures Acquisition and Consensus Clustering to Classify COAD Samples into EMT Subtypes

To obtain sufficient EMT scores for consensus clustering, we collected EMT genes from dbEMT2.0 (http://dbemt.bioinfo-minzhao.org/, (accessed on 4 September 2022) and the molecular signatures database of GSEA (http://www.gsea-msigdb.org/, (accessed on 4 September 2022). First, we separately performed average clustering on the EMT-related genes from the FOROUTAN_INTEGRATED_TGFB_EMT_UP, FOROUTAN_INTEGRATED_TGFB_EMT_DN, FOROUTAN_PRODRANK_TGFB_EMT_UP, FOROUTAN_PRODRANK_TGFB_EMT_DN, and FOROUTAN_TGFB_EMT_DN gene sets from the GSEA database, resulting in 15 sub-gene groups, with approximately 37 genes per gene set. We also used the gene set ALONSO_METASTASIS_EMT_UP from GSEA. For genes from the dbEMT website, we performed complete hierarchical clustering based on their expressions in the TCGA-COAD dataset and clustered the genes into five oncogenic EMT gene sets and five tumor-suppressive EMT gene sets, respectively. Finally, we obtained 26 EMT gene sets.

Based on the 26 EMT gene sets, we used the R package from GSVA (v 1.46.0) to obtain 26 EMT enrichment scores for each TCGA-COAD sample [16]. Subsequently, the enrichment score matrix was obtained. Then, based on the enrichment score of each sample, we performed cluster analysis by employing ConsensusClusterPlus [17] (v 1.62.0); the data were agglomerated in a cluster, and 80% of the samples were resampled ten times. After removing 5 samples without survival information and 24 samples with OS = 0 or 1, Kaplan–Meier analysis was performed on the selected 418 samples. 

### 4.3. EMT Marker Collection and Consensus Clustering to Verify the Clustering of 26 EMT Gene Sets

First, we collected 33 CRC-specific EMT marker genes from a prior CRC study (https://www.emtome.org, (accessed on 9 September 2022) [18]. Then, based on the gene expression profile, we performed cluster analysis by employing ConsensusClusterPlus in the TCGA-COAD dataset. Subsequently, we performed PCA using the R package stats. Using the ComplexHeatmap R package (v 2.14.0), we compared the expression heatmaps between the clustering of the 33 CRC EMT marker genes and the clustering of the 26 gene sets. We also calculated the correlation between the clusters from the two groups using the SubMap algorithm [19].

### 4.4. Construction of a Gene Co-Expression Network by WGCNA

To acquire EMT-associated gene modules more accurately and to correlate them with the phenotypes of the samples, we reobtained 499 samples from the TCGA-COAD dataset, including tumor and normal tissues, with 28,515 genes in the expression profile originally. Then, we used the WGCNA R package (v 1.72-1) to construct gene networks [20]. First, we utilized the gene expression profiles from the COAD dataset to calculate the median absolute deviation (MAD) of each gene and eliminated 50% of the genes of the lower MAD, with 14,258 genes remaining. Then, we used the goodSamplesGenes function and found no outlier genes or samples. Finally, we used the expression profile containing 499 samples and 14,258 genes to identify the gene co-expression network.

Next, using the Pearson’s product–moment correlation coefficient between genes, Pearson’s correlation matrices were constructed. Additionally, to determine the best soft thresholding (β), we obtained a weighted adjacency matrix by applying the function A_mn = |C_mn|^β (C_mn and A_mn are the Pearson’s correlation and the adjacency between Gene m and Gene n, respectively), and β was chosen to be 12. Based on the adjacency matrix, we built a topological overlap matrix (TOM), which displayed the average connectivity of the genes, and a correlative dissimilarity matrix (1-TOM). In addition, aiming to classify the interconnected genes into gene modules, we set 30 module genes as the minimum, and 4 as the sensitivity. After hierarchical clustering and the merging of some modules, 30 co-expression modules were obtained. Furthermore, we calculated the correlations between the gene expressions and phenotypic traits to determine gene significance (GS). Additionally, we calculated the module membership (MM) according to the relevance between the module genes and the gene expression profiles.

### 4.5. GO Analysis, KEGG Analysis, and PPI Network Construction

The selected module genes were analyzed by applying the clusterProfiler R package [21] (v 3.14.3), which provides Gene Ontology (GO) and Kyoto Encyclopedia of Genes and Genomes (KEGG) information.

The ComplexHeatmap R package [22] was utilized to show how hub genes in the lightcyan and darkolivegreen modules were expressed differentially. Then, the STRING database was used to identify the hub gene interactions in the protein–protein interaction (PPI) network.

### 4.6. Prognostic Model Construction Based on EMT Subtype-Associated Hub Genes

Using the survival R package (v 3.5-5) and integrating the OS time, survival status, and gene expression profiles of 585 module genes, we applied univariate Cox analysis to examine the value of each gene as a prognostic factor [23]. With the glmnet R package (v 4.1-7) applied, we then performed least absolute shrinkage and selection operator (LASSO) regression analysis and tenfold cross-validation to determine the best model [24]. Next, multivariate Cox analysis was performed using the random survival forest in the survival R package to screen for hub genes. Kaplan–Meier curves were then plotted using the survminer R package (v 0.4.9) to determine the prognostic significance of the hub genes [25].

Then, a combined risk score was computed based on the multivariable model using the following equation: risk score = ∑(βi×Expi), where *βi* indicated the Cox coefficient of the *i*th gene, and *Expi* indicated the expression value of the *i*th gene. Specifically, the risk score was calculated as follows: risk score = 0.974964442884743 × expression of *GPRC5B* − 1.30337826305244 × expression of *LSAMP* − 0.639834133401197 × expression of *PDGFRA*. Moreover, the ggplot2 R package (v 3.4.2) was implemented to visualize the relation between the gene expression levels, the risk score, and the OS status of the samples [26]. To evaluate the predictive performance of the risk score within 1, 3, and 5 years, we used the pROC R package (v 1.17.0.1) to carry out ROC analysis and obtained the area under the curve (AUC) values with their confidence intervals for each year [27].

### 4.7. Validation of the Prognostic Model

Using the survival R package and ggplot2, we accessed the prognostic value of the risk score using univariate and multivariate Cox analysis. An alluvial diagram was plotted by the ggalluvial R package (v 0.12.5) to show the distributions of COAD patients in different EMT clusters, their survival statuses, and the high- and low-risk groups. Then, with the rms R package (v 6.7-0) and survival R package applied, we plotted a nomogram and its calibration curves. 377 TCGA-COAD samples with TNM stage information were retained for the nomogram and risk-score related analysis. Moreover, to validate the prognostic value of the risk score, we plotted the Kaplan–Meier curves for the high- and low-risk groups from the TCGA-READ and GSE103479 dataset using the survminer R package.

### 4.8. TME Analysis

Immune cell infiltration was evaluated using Cell-type Identification by Estimating Relative Subsets of RNA Transcripts (CIBERSORT) [28] through the R package IOBR (v 0.99.9), and the abundance of immune cells was calculated for every sample. By using the Estimation of STromal and Immune cells in MAlignant Tumor tissues using Expression data (ESTIMATE)algorithm [29] of R package IOBR, we obtained the immune, stromal, and ESTIMATE scores for each TCGA-COAD sample.

### 4.9. Gene Mutation Analysis

We acquired somatic mutation data from the TCGA-COAD data portal. Then, by employing the maftools R package [30] (v 2.14.0), we made a waterfall plot to identify the frequently mutant genes in groups with high and low risk scores, respectively. The TMB score of each TCGA-COAD patient was computed by applying the maftools R package with the following equation: TMB score = (total mutations/total covered bases) × 10^6^.

### 4.10. Drug Sensitivity Predictions

We utilized TCGA-COAD samples to identify drugs that exhibited the differential effects between risk groups using the oncoPredict R package [31] (v 0.2), in combination with intestinal tissue cell lines from the Cancer Therapeutics Response Portal version 2 (CTRP v2) and Genomics of Drug Sensitivity in Cancer 2 (GDSC2) databases. We also calculated the half-maximal inhibitory concentrations (IC_50_) in the COAD samples using the ridge regression algorithms in the pRRophetic R package [32,33] (v 0.5). Additionally, we used tenfold cross-validation to evaluate the prediction accuracies. We also calculated the TIDE (tumor immune dysfunction and exclusion) scores with tidepy package [34] (v 1.3.8) (GitHub—jingxinfu/TIDEpy) of the TCGA-COAD samples to predict the immunotherapy responses.

### 4.11. Gene Set Enrichment Analysis (GSEA)

We used the GSEA software [35] (v 4.2.3) and chose the GO biological process (GOBP gene sets for analysis. Then, we analyzed and obtained the significantly enriched gene sets associated with the risk scores, with the size of the gene sets set to 5 to 5000. A *p*-value < 0.05 was considered statistically significant.

### 4.12. Statistical Analysis

Statistical analyses of the Kaplan–Meier curves were performed by log-rank test using the GraphPad Prism software (v 9.0.0). Other statistical analyses were conducted in the R software (v 4.2.2). The comparison between two paired groups were analyzed using Wilcoxon test and the comparison among three groups were analyzed using Kruskal–Wallis test. The categorical data were analyzed using χ^2^ test. The Pearson correlation coefficient was applied to measure the strength and direction of the relationship between two variables. A *p*-value < 0.05 was considered statistically significant.

## Data Availability

No new data were created.

## Supplementary Materials

The following supporting information can be downloaded at: https://www.mdpi.com/article/10.3390/ijms241713206/s1.
