# Supplementary material for: A Risk Model for Prognosis and Treatment Response Prediction in Colon Adenocarcinoma Based on Genes Associated with the Characteristics of the Epithelial-Mesenchymal Transition
Source: Int J Mol Sci. 2023 Aug 25;24(17):13206. doi: 10.3390/ijms241713206 (PMC10488217; doi:10.3390/ijms241713206)
Supplement: Supplementary file 1 [file ijms-24-13206-s001.zip › ijms-2496158-supplementary.pdf]

Supplementary Information

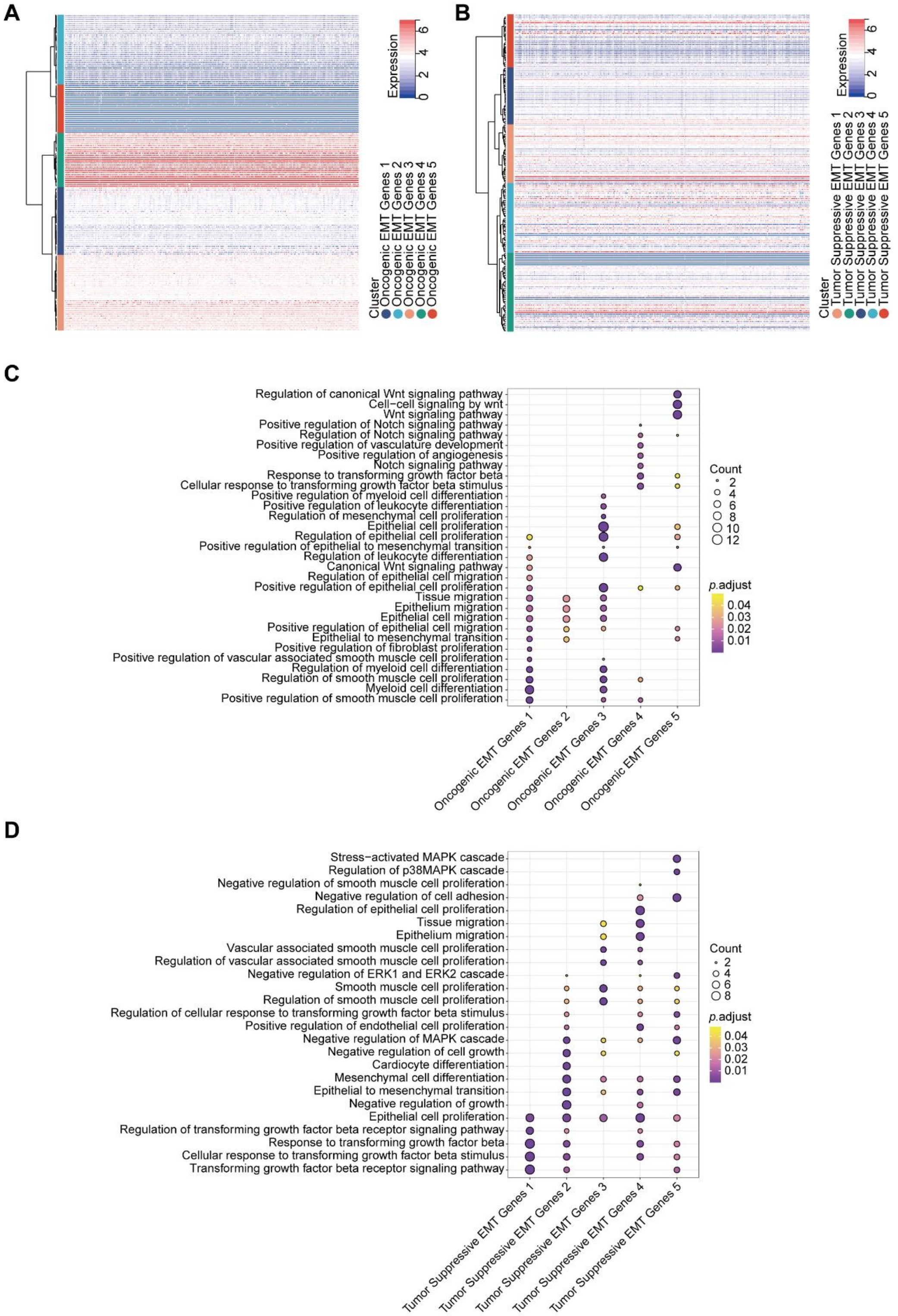

Figure S1. Complete hierarchical clustering of the epithelial-mesenchymal transition (EMT) genes from the dbEMT website. **A–B.** Heatmaps for the complete hierarchical clustering results of the oncogenic EMT genes (**A**) and the tumor suppressive EMT genes (**B**) obtained from dbEMT. **C–D.** Bubble plots of the enriched Gene Ontology (GO) pathways for the five oncogenic EMT gene sets (**C**) and five tumor-suppressive EMT gene sets (**D**).

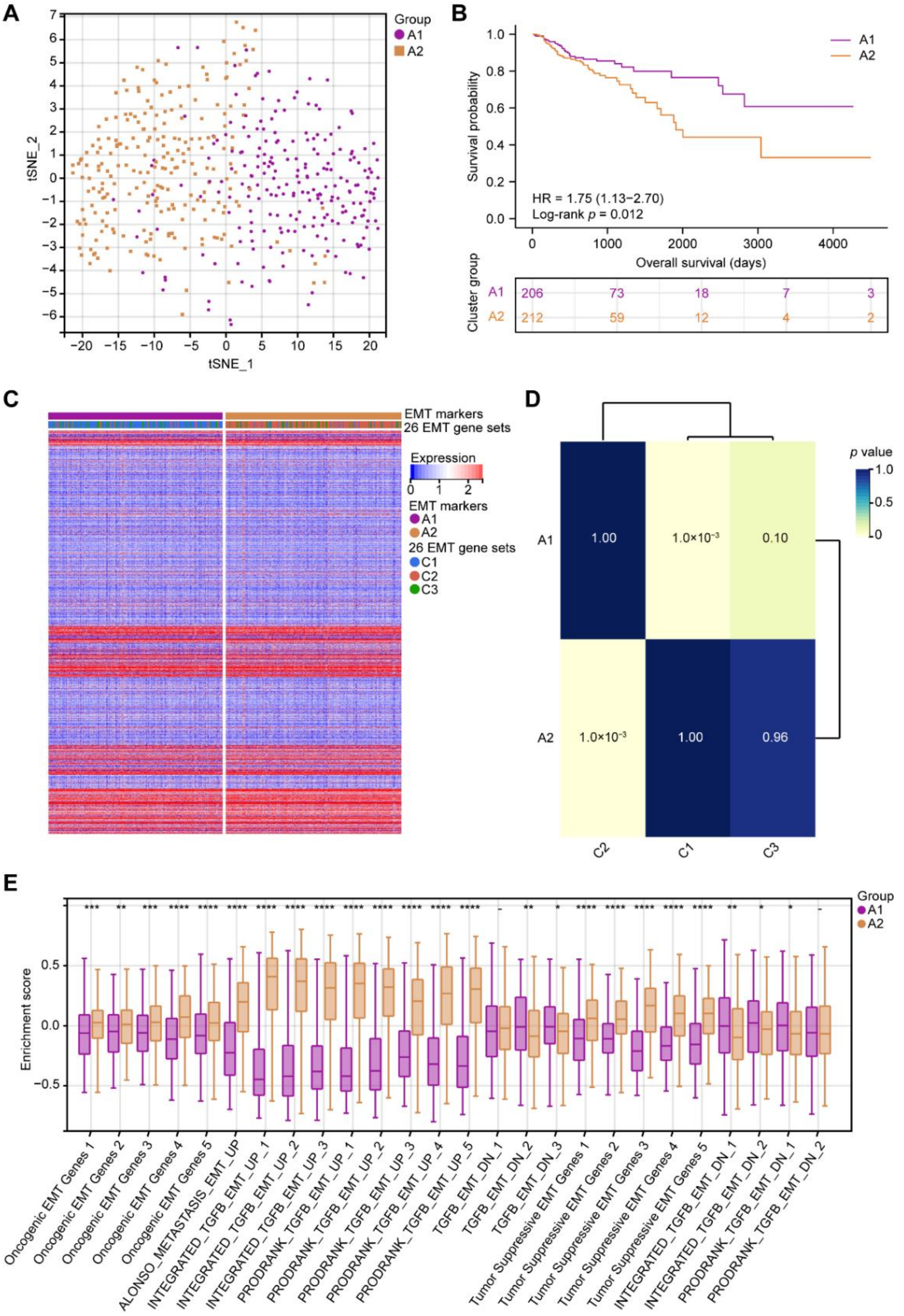

Figure S2. Validation of the three EMT clusters by an independent classification method. **A.** Principal component analysis (PCA) showing the differences between clusters A1 and A2. **B.** Kaplan–Meier overall survival (OS) curves for The Cancer Genome Atlas program (TCGA) colon adenocarcinoma (COAD) patients categorized as clusters A1 and A2. **C.** Heatmap showing the gene expression data in A1, A2, C1, C2, and C3 clusters. **D.** Submap showing the correlation significance (adjusted nominal *p* by Fisher's test) between the different clusters. **E.** Box plot of GSVA enrichment scores for 26 EMT gene sets in clusters A1 and A2. Wilcoxon test; -, no significance, $*p < 0.05$, $**p < 0.01$, $***p < 0.001$, $****p < 0.0001$.

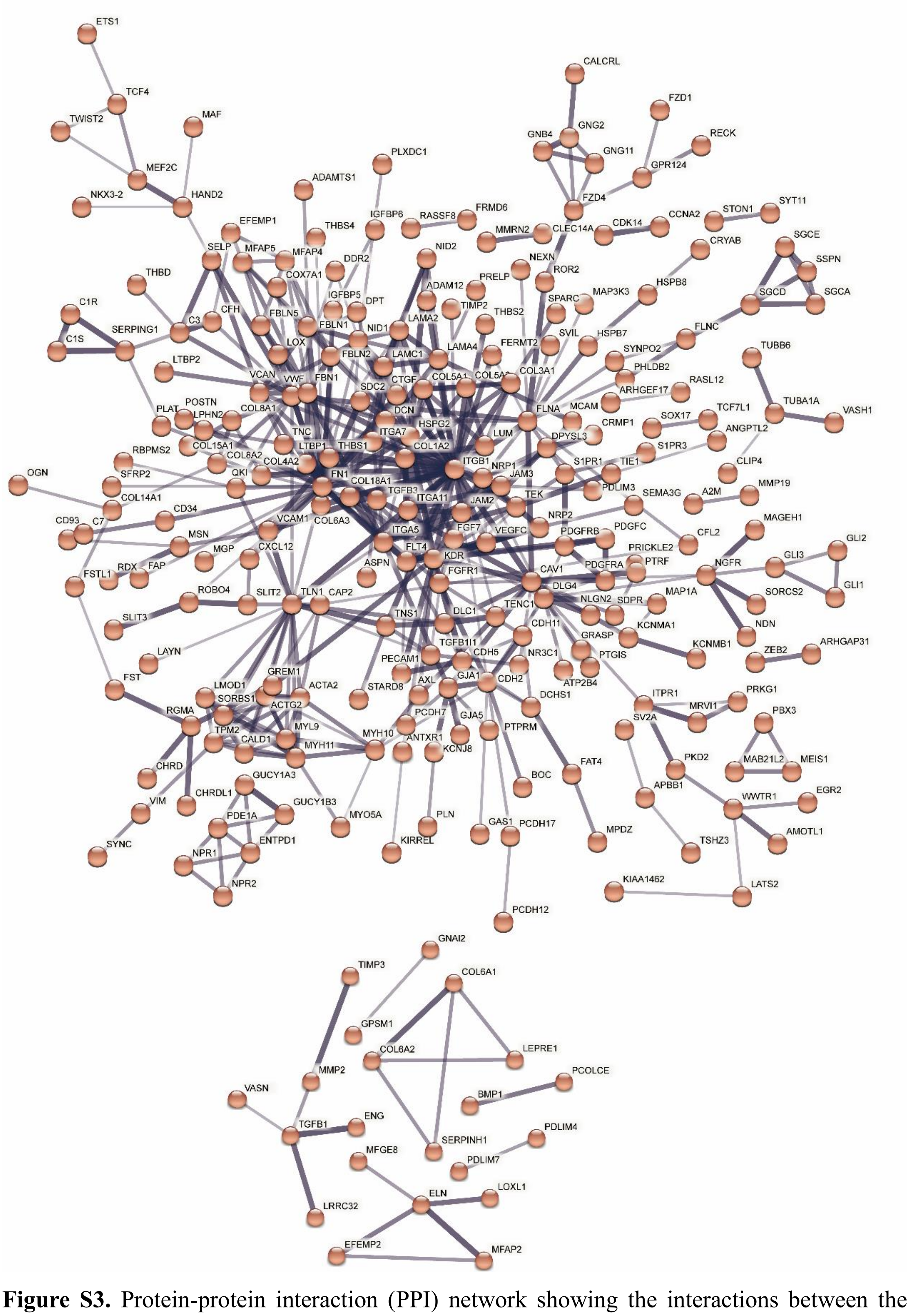

Figure S3. Protein-protein interaction (PPI) network showing the interactions between the genes from the lightcyan and darkolivegreen modules.

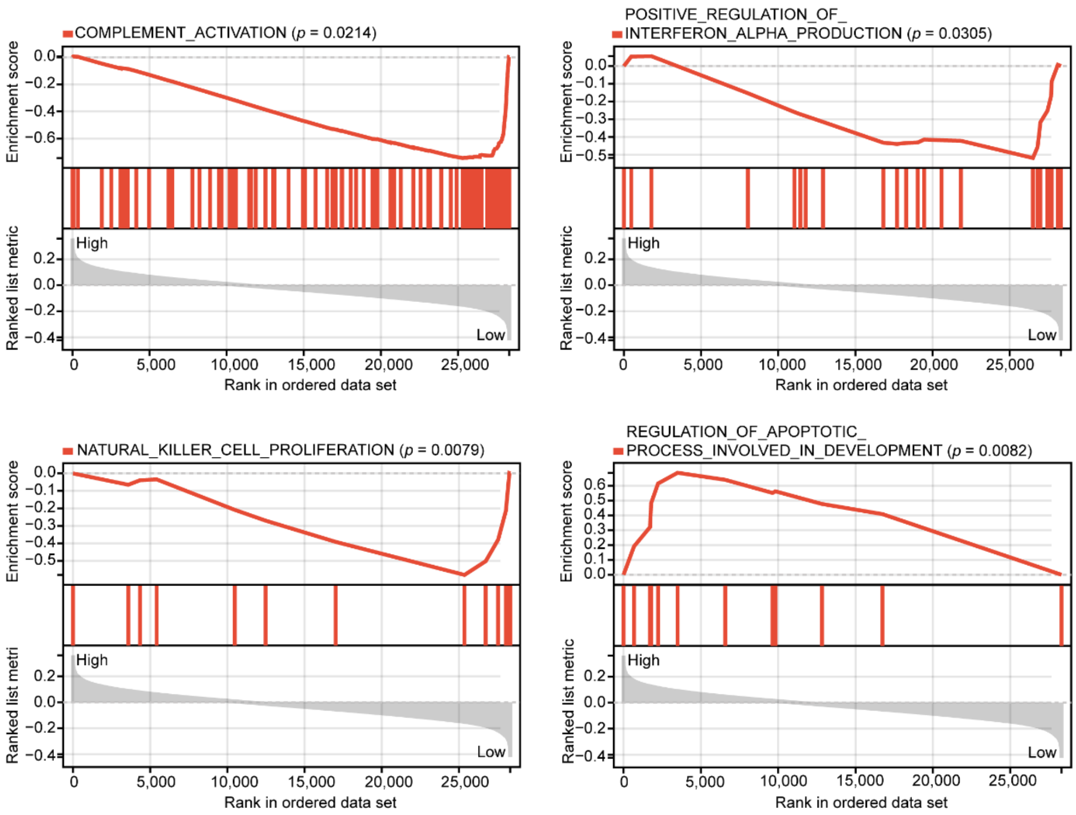

Figure S4. GSEA analysis of different GO biological process gene sets associated with the risk score.

Table S1. The ten EMT gene sets acquired from dbEMT database.

Gene set	Gene								
Oncogenic EMT Genes 1	*RAB22A*	*HOXA9*	*JUNB*	*RAF1*	*EPCAM*	*HRAS*	*WWTR1*	*CDK14*	*SOX4*	*SETDB1*
	MYB	*PIM2*	*JUN*	*KRAS*	*CEACAM6*	*MYD88*	*JAK2*	*FASN*	*TBX3*	*MIR221*
	PDGFRB	*ETS1*	*S100A4*	*GATA6*	*MIR10B*	*FOXQ1*	*PIK3CA*	*FZD2*	*TAZ*	*FOXM1*
	AKT2	*LCN2*	*PLAC8*	*FHL2*	*MYCN*	*CUL4A*	*NEAT1*			
Oncogenic EMT Genes 2	*MIR191*	*SNAI1*	*MIR93*	*MET*	*ETV4*	*BRF2*	*FGFR1*	*ZEB1-AS1*	*MALAT1*	*MIR485*
	ELK1	*TBX2*	*TXN*	*BRD4*	*PRKCI*	*SQSTM1*	*EIF5A2*	*ZNF217*	*ACTN4*	*MIR663A*
	ERG	*TYMS*	*CD24*	*IRS2*	*AKTIP*	*ITGA3*	*NANOG*	*PPM1D*	*PTP4A2*	*CAMK1D*
	TRIM28	*MIR520G*	*ROS1*	*CIP2A*	*UHRF1*	*ROCK1*	*TFCP2*			
Oncogenic EMT Genes 3	*CTNNB1*	*SREBF1*	*LEF1*	*GNA13*	*MDM2*	*YY1*	*PAX2*	*AXL*	*ID1*	*PRKCA*
	BRAF	*YWHAZ*	*NCOA3*	*PDGFB*	*EEF1D*	*FOSL1*	*HOTAIR*	*MITF*	*BCL2L1*	*SIX1*
	ID2	*ELAVL1*	*UCA1*	*YBX1*	*MIR135B*	*MUC1*	*PAK5*	*MYC*	*PTP4A3*	*KDM5B*
	MIR92B	*ERBB2*	*HOXD9*	*MIR373*	*RHOC*	*MIR96*	*LIN28A*	*CRK*	*MIR506*	*S100A8*
Oncogenic EMT Genes 4	*CREB1*	*GOLPH3*	*SKI*	*GLI2*	*AKT1*	*EIF3I*	*MTDH*	*ARHGEF2*	*GSK3A*	*PAK1*
	MIR301A	*ALK*	*HSPB1*	*CXCR4*	*YWHAG*	*MCL1*	*IGF1R*	*MDM4*	*MAP3K7*	*TWIST1*
	USP22	*EPS8*	*GLO1*	*AR*	*BIRC2*	*NANOGP8*	*SATB1*	*LETMD1*	*EIF4E*	*KIT*
	WNT1	*NOTCH4*	*MACC1*	*CRKL*	*UBE3C*	*BMI1*				
Oncogenic EMT Genes 5	*FGFR2*	*TLE1*	*AURKA*	*ETV1*	*RAC1*	*BCL2*	*MUC4*	*HSPA5*	*GAB2*	*ELK3*
	CBLB	*BIRC5*	*MIR454*	*ALDH1A1*	*KLF8*	*FRAT1*	*BMP7*	*CCND1*	*GMNN*	*HSPA4*
	EGFR	*SOX2*	*MIR128-1*	*EML4*	*MTOR*	*MLLT3*	*CRYAB*	*SRC*	*PLAGL2*	
Tumor Suppressive EMT Genes 1	*CREBBP*	*GPC3*	*NUMB*	*TP53BP2*	*AFAP1L2*	*LOX*	*HDAC3*	*VDR*	*NR1I2*	*CEACAM1*
	CTNNBIP1	*IGFBP3*	*IGFBP7*	*VHL*	*YPEL3*	*CTNND1*	*EPHB3*	*LIMA1*	*RUNX2*	*HPGD*
	ABCG2	*HIPK2*	*RNH1*	*NEDD4L*	*SIRT3*	*DKK3*	*SIAH1*	*GSN*	*KDM5A*	*INPP4B*
	RB1	*LEFTY1*	*PCGF2*	*SMAD2*	*ANXA1*	*DAB2*	*ARHGEF12*	*PHLDA2*	*ZYX*	
Tumor Suppressive EMT Genes 2	*ESR2*	*NOTCH2*	*SCUBE2*	*ISG15*	*BBC3*	*SMAD4*	*PRKAA1*	*TXNIP*	*STK11*	*AHR*
	CLDN1	*TCF4*	*CDH11*	*FBP1*	*BMP4*	*DKK1*	*AXIN2*	*ESRRB*	*TGFBR3*	*EGLN3*
	TGFBR2	*TSC1*	*HNF4A*	*STAT5A*	*SLC9A3R1*	*S100A2*	*CLU*	*BATF2*	*PEBP1*	*KDM6A*
	NR4A1	*GSK3B*	*EPAS1*	*GKN2*	*HRG*	*ROR2*	*CD44*	*PDCD4*	*IL17A*	*ITGA5*
Tumor Suppressive EMT Genes 3	*MTUS1*	*CXCR2*	*NR2C2*	*NUAK1*	*RACK1*	*STAT1*	*TNFAIP8L2*	*AGTR1*	*PKD1*	*NDRG2*
	ESR1	*ATM*	*FBXW7*	*GJB2*	*FOXC1*	*FOXO4*	*KDM3A*	*WWOX*	*CD82*	*ITGB3*
	DAPK1	*KL*	*RHOB*	*DNMT1*	*PCDH9*	*SUFU*	*WNT11*	*IGF1*	*TDGF1*	*PTPN6*
	LATS1	*FHL1*	*CSF2*	*FAS*	*DICER1*	*BRD7*	*DDR2*	*FBLN1*	*VSNL1*	
Tumor Suppressive EMT Genes 4	*UIMC1*	*EPB41L3*	*HOXB13*	*TWIST2*	*ING4*	*PARP1*	*SPOP*	*DNMT3B*	*BRMS1*	*RASAL2*
	NF1	*CXCL12*	*CCNDBP1*	*SCRIB*	*PROX1*	*HIF1A*	*ERRFI1*	*KLK6*	*ITGB1*	*FOXO3*
	ESRP1	*FLNA*	*FOXA2*	*LRIG1*	*KRT19*	*SOCS3*	*AXIN1*	*PRKAA2*	*ZFP36*	*MARVELD1*
	TNFSF12	*TSC2*	*THBD*	*TP53*	*CDH5*	*RNF8*	*SOD2*	*BRCA1*	*EGR1*	*PTEN*
Tumor Suppressive EMT Genes 5	*IL17RD*	*VEGFA*	*UHRF2*	*ANGPTL4*	*PAWR*	*BMP2*	*RNF111*	*DAB2IP*	*NFKB1*	*PIN1*
	MAP3K4	*TIMP3*	*SFRP2*	*GLS2*	*TP53BP1*	*TP53INP1*	*TRIM62*	*IFT88*	*TUSC7*	*CDKN2A*
	MEG3	*EPHB2*	*CDH13*	*ERF*	*AJAP1*	*EED*	*NOTCH3*	*NDRG1*	*IRF8*	*CXCL14*
	CFTR	*HIC1*								

Table S2. The sixteen EMT gene sets acquired from GSEA database.

Gene set	Gene									
ALONSO_	*ANLN*	*APLP2*	*CD63*	*CDH2*	*CLIC4*	*CTSB*	*CX3CR1*	*DSG2*	*EDNRB*	*EMP1*
METASTASIS_EMT_UP	*ENC1*	*FGG*	*FZD1*	*HMMR*	*ITGAV*	*L1CAM*	*LUM*	*MFAP1*	*MMP2*	*PFN1*
	PRKCA	*RAB1A*	*RAN*	*RRAGA*	*SDCBP*	*SELENOP*	*SERPINA3*	*SMARCA1*	*SPA17*	*SPARC*
	TUBA1A	*TUBA3C*	*TUBA4A*	*TUBB3*	*VCAN*					
INTEGRATED_	*ACKR3*	*ADAM19*	*ADAMTS6*	*ALOX5AP*	*ANGPTL4*	*AP1S2*	*ARHGEF40*	*BMPR2*	*CALD1*	*CCN2*
TGFB_EMT_UP_1	*CDH11*	*CDH2*	*CDK14*	*CHRNA9*	*COL1A1*	*COL3A1*	*COL4A1*	*COL4A2*	*COL5A1*	*COL5A2*
	COL6A3	*COL7A1*	*CRLF1*	*DACT1*	*DHRS2*	*DIXDC1*	*DLC1*	*DOCK4*	*DSE*	*EPHB2*
	FBN1	*FERMT2*	*FHOD3*	*FN1*	*FOXD1*	*FSTL3*	*GADD45B*	*GAL*	*GALNT10*	
INTEGRATED_	*GASK1B*	*GFPT2*	*GLIPR1*	*GREM1*	*HMOX1*	*HS3ST3A1*	*HS3ST3B1*	*HSF2BP*	*HTRA1*	*IGFBP5*
TGFB_EMT_UP_2	*IGFBP7*	*IL11*	*INHBA*	*ITGA5*	*ITGB3*	*JUN*	*JUNB*	*KCNMA1*	*LMCD1*	*LOX*
	LUM	*MAF*	*MFAP2*	*MMP1*	*MMP10*	*MMP2*	*MN1*	*MRC2*	*MYL9*	*NCF2*
	NEDD9	*NKX3-1*	*NREP*	*NT5E*	*NUAK1*	*PDGFC*	*PDLIM7*	*PID1*	*PLEK2*	
INTEGRATED_	*PMEPA1*	*PODXL*	*POSTN*	*PTHLH*	*PTPRK*	*RGS4*	*SACS*	*SCG2*	*SCG5*	*SERPINE1*
TGFB_EMT_UP_3	*SERPINE2*	*SKIL*	*SLC22A4*	*SLC26A2*	*SLN*	*SMAD7*	*SNAI2*	*SPARC*	*SPHK1*	*SPOCK1*
	SRPX	*STC1*	*TAGLN*	*TCF4*	*TGFB1*	*TGFB1I1*	*TGFBI*	*TGM2*	*THBS1*	*TIMP2*
	TNFAIP6	*TNS1*	*TP53I3*	*TPM1*	*TUFT1*	*VCAN*	*WNT5A*	*WNT5B*	*XYLT1*	*ZNF365*
PRODRANK_	*ABCA1*	*ACKR3*	*ACTN1*	*ADAM12*	*ADAM19*	*ADAMTS6*	*AKT3*	*ALOX5AP*	*AMIGO2*	*ANGPTL4*
TGFB_EMT_UP_1	*ANKLE2*	*AP1S2*	*APBB2*	*ARFGAP1*	*ARHGEF40*	*ARNTL*	*BHLHE40*	*BMP1*	*BMP2*	*BMPR2*
	BPGM	*C3orf52*	*CALD1*	*CCN2*	*CD59*	*CDH11*	*CDH2*	*CDK14*	*CHRNA9*	*CHST11*
	COL1A1	*COL4A1*	*COL4A2*	*COL5A1*	*COL7A1*	*CRLF1*	*CYTH1*			
PRODRANK_	*SNAI2*	*SPARC*	*SPDL1*	*SPHK1*	*SPOCK1*	*SRPX*	*SRRD*	*STC1*	*TAGLN*	*TAGLN2*
TGFB_EMT_UP_2	*TBX3*	*TCF4*	*TFPI2*	*TGFB1*	*TGFB1I1*	*TGFBI*	*TGM2*	*THBS1*	*TIMP2*	*TMCC1*
	TNFAIP6	*TNS1*	*TP53I3*	*TPM1*	*TPM4*	*TPST1*	*TPST2*	*TUBA1A*	*TUBA4A*	*TUFT1*
	VCAN	*VEGFC*	*VIM*	*WNT5A*	*WNT5B*	*XYLT1*	*ZNF365*			
PRODRANK_	*NT5E*	*NUAK1*	*PALLD*	*PDGFA*	*PDGFC*	*PDLIM7*	*PEA15*	*PID1*	*PIK3CD*	*PLAUR*
TGFB_EMT_UP_3	*PLEK2*	*PMEPA1*	*PODXL*	*POSTN*	*PRR5L*	*PSMD2*	*PTHLH*	*PTPN21*	*PTPRK*	*PXDC1*
	RALA	*RFTN1*	*RGS4*	*RUNX2*	*SACS*	*SCG5*	*SEMA3C*	*SERPINE1*	*SERPINE2*	*SIK1*
	SKIL	*SLC22A4*	*SLC26A2*	*SLCO2A1*	*SLN*	*SMAD7*	*SMURF2*			
PRODRANK_	*INHBA*	*INPP4B*	*ITGA5*	*ITGB3*	*JAG1*	*JARID2*	*JUN*	*JUNB*	*KCNJ15*	*KCNMA1*
TGFB_EMT_UP_4	*KDELR3*	*KLF7*	*LAMC2*	*LARP6*	*LBH*	*LMCD1*	*LOX*	*MAF*	*MAGED2*	*MAP1LC3B*
	MATN3	*MBOAT2*	*MFAP2*	*MICAL2*	*MMP1*	*MMP10*	*MMP2*	*MMP9*	*MN1*	*MRC2*
	MYL9	*MYO10*	*NCF2*	*NEDD9*	*NKX3-1*	*NREP*	*NRIP3*			
PRODRANK_	*DAAM1*	*DACT1*	*DHRS2*	*DIXDC1*	*DLC1*	*DOCK4*	*DSE*	*DUSP10*	*ELK3*	*EML1*
TGFB_EMT_UP_5	*EPHB2*	*ETS2*	*FAM114A1*	*FBN1*	*FERMT2*	*FHOD3*	*FN1*	*FOXD1*	*FSTL3*	*GADD45B*
	GAL	*GALNT10*	*GASK1B*	*GFPT2*	*GLIPR1*	*GNG11*	*GRB10*	*GREM1*	*HMGA2*	*HMOX1*
	HRH1	*HS3ST3A1*	*HSF2BP*	*HTRA1*	*IGFBP5*	*IGFBP7*	*IL11*			
TGFB_EMT_DN_1	*ABLIM1*	*ADORA2B*	*AGR2*	*ALDH1A3*	*ALDH3A2*	*ALDH5A1*	*ANK3*	*AQP3*	*AREG*	*ARHGAP29*
	ATP8B1	*BIRC3*	*C1orf115*	*C1orf116*	*CAVIN2*	*CD9*	*CDH1*	*CEACAM6*	*CEBPD*	*CFB*

	CFH	*CITED2*	*CP*	*CXADR*	*CYB5A*	*CYP1B1*	*DEFB1*	*DEPTOR*	*DST*	*EHF*
	ELF3	*EMP1*	*EPAS1*	*EPB41L4B*	*EPCAM*	*ERBB3*				
TGFB_EMT_DN_2	*NUP210*	*PDK4*	*PEG10*	*PKP2*	*PLAAT3*	*PLAAT4*	*PLS1*	*PPL*	*PPP1R9A*	*RAB25*
	RAB26	*RAB38*	*RBM47*	*S100P*	*SCNN1A*	*SERPINB1*	*SLC16A7*	*SLC27A2*	*SLCO4A1*	*SLPI*
	SMPDL3B	*SORD*	*SOX2*	*SPRY1*	*SQOR*	*SULT1A1*	*SYBU*	*SYNE2*	*TBC1D8*	*TFAP2A*
	TJP2	*TMEM30B*	*TNFAIP2*	*TPD52L1*	*TSPAN1*	*VAV3*				
TGFB_EMT_DN_3	*EREG*	*ERMP1*	*ESRP1*	*FA2H*	*FGFBP1*	*FOXA2*	*GDF15*	*GLDC*	*GPRC5C*	*GRTP1*
	GSE1	*GULP1*	*HOOK1*	*HPGD*	*HS3ST1*	*IMPA2*	*INHBB*	*JAG2*	*KITLG*	*KRT15*
	KRT19	*LAMA5*	*LCN2*	*LSR*	*LY6E*	*MANSC1*	*MAP7*	*MBP*	*METTL7A*	*MITF*
	MMP7	*MPZL2*	*MTUS1*	*MUC1*	*MYO5C*	*NR2F2*				
INTEGRATED_	*HPGD*	*HS3ST1*	*IMPA2*	*INHBB*	*KRT15*	*KRT19*	*LCN2*	*LSR*	*LY6E*	*MANSC1*
TGFB_EMT_DN_1	*MBP*	*METTL7A*	*MMP7*	*MPZL2*	*MTUS1*	*MYO5C*	*PDK4*	*PKP2*	*PLAAT4*	*PLS1*
	PPL	*PPP1R9A*	*RAB25*	*RBM47*	*S100P*	*SCNN1A*	*SERPINB1*	*SLC16A7*	*SLC27A2*	*SLPI*
	SOX2	*SYNE2*	*TBC1D8*	*TJP2*	*TMEM30B*	*TSPAN1*	*VAV3*			
INTEGRATED_	*AGR2*	*ALDH1A3*	*ANK3*	*AQP3*	*AREG*	*ARHGAP29*	*ATP8B1*	*BIRC3*	*C1orf116*	*CAVIN2*
TGFB_EMT_DN_2	*CD9*	*CDH1*	*CEACAM6*	*CEBPD*	*CFB*	*CFH*	*CP*	*CXADR*	*CYB5A*	*DEFB1*
	DEPTOR	*DST*	*EHF*	*ELF3*	*EMP1*	*EPB41L4B*	*EPCAM*	*ERBB3*	*EREG*	*ERMP1*
	ESRP1	*FA2H*	*FGFBP1*	*FOXA2*	*GDF15*	*GRTP1*	*GSE1*			
PRODRANK_	*ABLIM1*	*ADORA2B*	*AGR2*	*ALDH3A2*	*ALDH5A1*	*ANK3*	*AREG*	*ARHGAP29*	*BIRC3*	*C1orf115*
TGFB_EMT_DN_1	*C1orf116*	*CAVIN2*	*CD9*	*CDH1*	*CEBPD*	*CITED2*	*CXADR*	*CYB5A*	*CYP1B1*	*DEPTOR*
	DST	*EHF*	*ELF3*	*EPAS1*	*EPB41L4B*	*EPCAM*	*ERBB3*	*EREG*	*ERMP1*	*ESRP1*
	FOXA2	*GDF15*	*GLDC*	*GPRC5C*	*GSE1*	*GULP1*	*HOOK1*	*HPGD*	*IMPA2*	*JAG2*
	KITLG									
PRODRANK_	*KRT19*	*LAMA5*	*LY6E*	*MAP7*	*MBP*	*METTL7A*	*MITF*	*MMP7*	*MTUS1*	*MUC1*
TGFB_EMT_DN_2	*MYO5C*	*NR2F2*	*NUP210*	*PDK4*	*PEG10*	*PKP2*	*PLAAT3*	*PLAAT4*	*PLS1*	*PPL*
	PPP1R9A	*RAB26*	*RAB38*	*RBM47*	*S100P*	*SCNN1A*	*SERPINB1*	*SLC16A7*	*SLC27A2*	*SLCO4A1*
	SMPDL3B	*SORD*	*SPRY1*	*SQOR*	*SULT1A1*	*SYBU*	*TBC1D8*	*TFAP2A*	*TJP2*	*TNFAIP2*
	TPD52L1									
